# A novel seed dispersal mode of *Apostasia nipponica* could provide some clues to the early evolution of the seed dispersal system in Orchidaceae

**DOI:** 10.1002/evl3.188

**Published:** 2020-08-02

**Authors:** Kenji Suetsugu

**Affiliations:** ^1^ Department of Biology Graduate School of Science Kobe University Kobe Hyogo 657–8501 Japan

**Keywords:** Apostasioideae, camel cricket, cricket, endozoochory, Orchidaceae, seed disperser

## Abstract

Despite being one of the most diverse families, scant attention has been paid to the seed dispersal system in Orchidaceae, owing to the widely accepted notion that wind dispersal is the dominant strategy. However, the indehiscent fruits, with seeds immersed in fleshy tissue, evoke the possibility of endozoochory in Apostasioideae, the earliest diverging lineage of orchids. In the present study, I investigated the seed dispersal system of *Apostasia nipponica* by direct observation, time‐lapse photography, and investigation of the viability of seeds passing through the digestive tract of orthopterans. This study revealed a previously undocumented seed dispersal system in *A. nipponica*, in which the cricket, *Eulandrevus ivani*, and the camel cricket, *Diestrammena yakumontana*, consume the fruit and defecate viable seeds. Orthopterans are rarely considered seed dispersers, but the gross fruit morphology and pigmentation patterns of some *Apostasia* species parallel those seen in *A. nipponica*, suggesting that similar seed dispersal systems could be widespread among *Apostasia* species. Whether seed dispersal by orthopteran frugivores is common in Apostasioideae warrants further investigation.

**Impact summary**

Seed dispersal is a key evolutionary process and central theme in terrestrial plant ecology. Animal‐mediated seed dispersal, most frequently by birds and mammals, benefits seed plants by ensuring efficient and directional transfer of seeds without relying on random abiotic factors such as wind and water. In return for these seed dispersal services, many plants provide nutritional rewards in the form of fleshy fruits. Orchids are unique among terrestrial plants in that their seedlings are completely dependent on fungi for their nutritive needs until they are mature enough to photosynthesize. Therefore, orchids usually produce remarkably small seeds that lack endosperm and are dispersed in air like dust particles. Therefore, the prevailing assumption is that orchid seeds are dispersed by wind. However, because indehiscent fruits with hard seed coats are common within the subfamily Apostasioideae, the earliest diverging clade in Orchidaceae, animal‐mediated seed dispersal can be an ancestral trait in the clade. Here, I present evidence for seed dispersal by crickets and camel crickets in *Apostasia nipponica* (Apostasioideae). To my knowledge, this is the first detailed report of a seed dispersal system in Apostasioideae. Owing to many plesiomorphic characters and the earliest diverging phylogenetic position, members of Apostasioideae have been extensively studied to understand their floral structure, taxonomy, biogeography, and genome. The interaction described here, importantly, provides some clues to the animals that may have participated in the seed dispersal of the ancestors of orchids. Given that the origin of crickets and camel crickets precedes the evolution of orchids, they are among the candidates for seed dispersers of the ancestors of extant orchids. Ancestral character‐state reconstruction analysis, with more data on the seed dispersal systems of other apostasioids, can provide deeper insights into the early evolution of the seed dispersal system in Orchidaceae.

Orchidaceae is one of the largest and most diverse families of flowering plants, with more than 28,000 known species spanning 763 genera (Christenhusz and Byng 2016). This high species diversity is likely linked to its specialized pollination syndromes, symbiotic associations with mycorrhizal fungi, colonization of epiphytic habitats, tropical and cordillera distribution, and its use of crassulacean acid metabolism (Givnish et al. 2015; Zhang et al. 2018). Consequently, researchers have paid considerably more attention to orchid pollination and orchid‐mycorrhizal symbiosis than to orchid seed dispersal (Zhang et al. 2018), even though orchids also exhibit diverse fruit morphology (Dirks‐Mulder et al. 2019).

Limited attention has been paid to the mode of orchid seed dispersal, probably owing to the dogma that wind seed dispersal is the dominant strategy (Arditti and Ghani 2000). Orchid seeds are very small and extremely light, and are produced in large numbers (Jersáková and Malinová 2007). These seeds do not possess an endosperm but instead usually have large internal air spaces that allow them to float in the air column (Arditti and Ghani 2000). In addition, orchid seeds are usually winged or filiform, evolved to be potentially carried with air currents (Kiyohara et al. 2012; Fan et al. 2020). Furthermore, most orchid seeds have thin papery coats formed by a single layer of nonlignified dead cells (Molvray and Chase 1999). It has been considered that the fragile thin seed coats cannot withstand the digestive fluids of animals (Garay 1964; McCormick et al. 2013), in contrast to the thick seed coats in indehiscent fruits, which are considered an adaptation for endozoochory (Jordano 1995).

Although indehiscent fruits and seeds with hard seed coats are rare in Orchidaceae, they can be found in some species of several subfamilies. Using molecular studies, orchids have been divided into five subfamilies: Apostasioideae, Vanilloideae, Cypripedioideae, Orchidoideae, and Epidendroideae (Givnish et al. 2015). Indehiscent fruits with seeds covered with a hard seed coat (Arditti and Ghani 2000; Molvray and Chase 1999) include those of *Selenipedium* (Cypripedioideae), *Vanilla* and *Cyrtosia* (Vanilloideae), and *Palmorchis* and *Yoania* (Epidendroideae). Among these orchids, *C. septentrionalis* and two *Yoania* species are dispersed by frugivorous birds and camel crickets, respectively (Suetsugu et al. 2015; Suetsugu 2018a,b).

Notably, the subfamily Apostasioideae commonly has indehiscent fruits with hard, crustose black seed coats (Molvray and Chase 1999). Apostasioids are the earliest diverging subfamily of orchids and consist of only two genera (*Apostasia* and *Neuwiedia*), with only about 20 species distributed in southeastern Asia, Japan, and northern Australia (Chen et al. 2017). All *Apostasia* and most *Neuwiedia* species investigated to date are known to possess berries with hard seed coats (Kocyan and Endress 2001; Molvray and Chase 1999). Consequently, it has been suspected that the fruits are consumed by animals (Clements 1999). In addition, some other traits, such as inconspicuous fruits at the ground level, might be associated with endozoochory by terrestrial invertebrates, given that camel crickets disperse the seeds of several mycoheterotrophic plants with similar fruit presentation (Suetsugu 2018a,b). In fact, orthopteran visitors (i.e., crickets and camel crickets) were observed feeding on the ripe fruits of *Apostasia nipponica* in my preliminary field investigation.

Apostasioids are well known for several unique traits, such as a nonresupinate flower with an actinomorphic perianth and pollen grains that do not form pollinia (Kocyan and Endress 2001; Zhang et al. 2017), although they also share some synapomorphies with other orchids, such as small seeds with a reduced embryo and mycoheterotrophic protocorms (Kristiansen et al. 2001). These characters have been considered ancestral in orchids, given that they are similar to those found in the members of Hypoxidaceae (which is closely related to Orchidaceae; Zhang et al. 2017). Similarly, the presence of an indehiscent fruit with a thick seed coat, found in most *Apostasia* and *Neuwiedia* species, may be plesiomorphic in Orchidaceae (Molvray and Chase 1999). Intriguingly, Orthoptera is one of the oldest insect orders, and its fossil records are available from the Late Carboniferous era (Gorochov et al. 2006); the origin of crickets and camel crickets predates the evolution of orchids (Givnish et al., 2015, 2016; Song et al. 2015). Therefore, crickets and camel crickets are arguably among the candidate seed dispersers for the ancestors of extant orchids, if they defecate viable seeds.

In the present study, I investigated the seed dispersal system of *Apostasia nipponica* to demonstrate a potential mutualism between crickets/camel crickets and *A. nipponica*. Specifically, I investigated whether (i) *A. nipponica* fruits are mainly consumed by crickets and camel crickets through direct observation and time‐lapse photography, (ii) the seeds defecated by crickets and camel crickets remain viable by checking their viability by 2,3,5‐triphenyl tetrazolium chloride (TTC) staining, and (iii) the fruits and seeds of *A. nipponica* are adapted for dispersal by animals through anatomical investigations using microtome sectioning to demonstrate endozoochory between crickets/camel crickets and *A. nipponica*.

## Material and Methods

### FIELD STUDY

**Figure 1:**
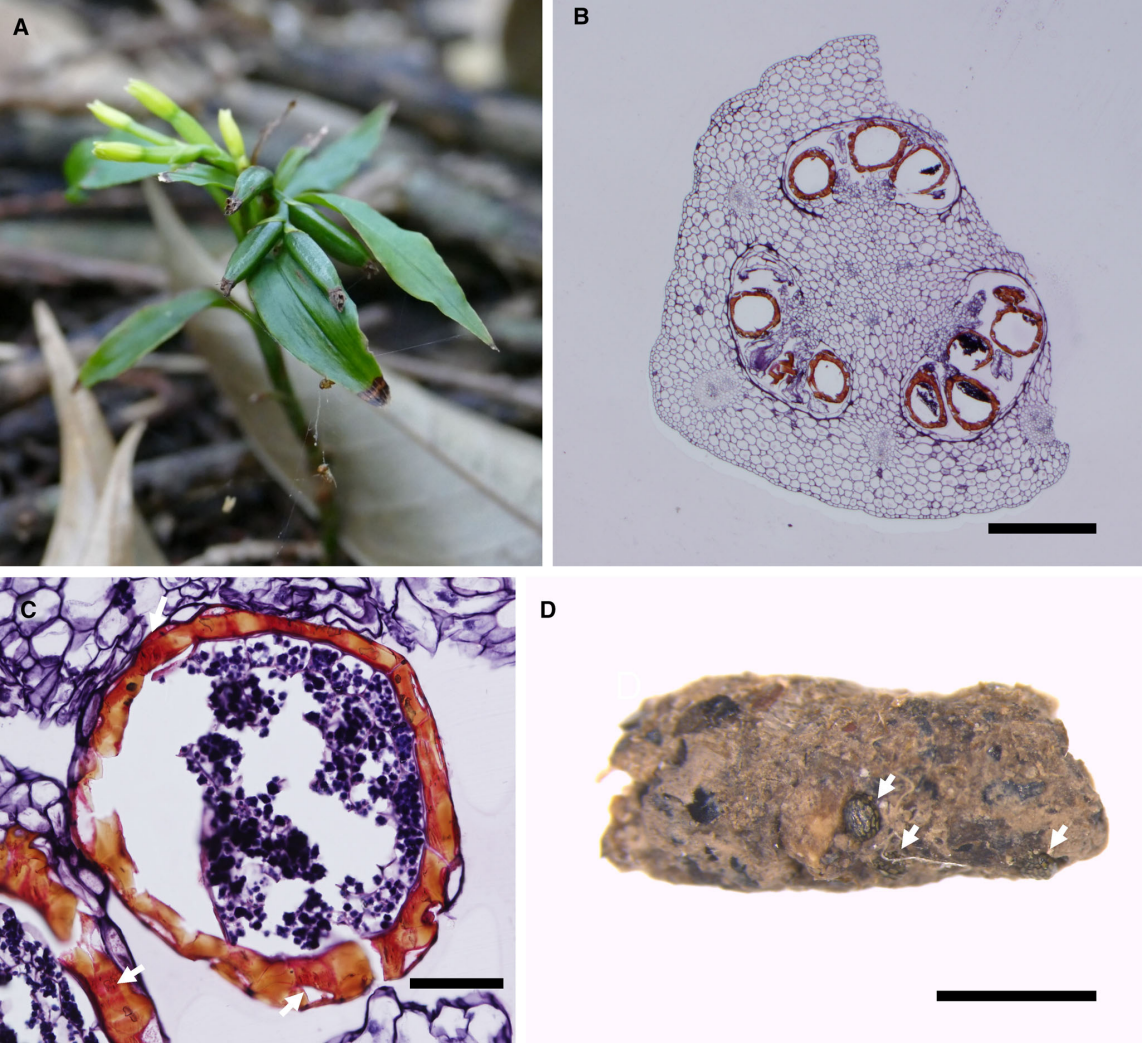
(A) An *Apostasia nipponica* plant. (B) Cross section of an *A. nipponica* fruit (Scale bar = 500 μm). (C) Cross section of an *A. nipponica* seed (Scale bar = 50 μm). The thickened lignified tissues stained by Safranin O were indicated by arrows. (D) Cricket *Eulandrevus ivani* feces containing *A. nipponica* seeds (indicated by arrows) (Scale bar = 1 mm).

*Apostasia nipponica* develops fleshy, succulent, indehiscent fruits that mature approximately 12 months after flowering (Fig. 1).

Field studies were carried out on Yakushima Island, Kagoshima Prefecture, Japan. The study site contained approximately 10 fruiting individuals of *A. nipponica*, with each plant having one to four mature fruits. The population size in the present study was slightly limited because *A. nipponica* is very rare throughout its distribution area and its population size is typically very small. Direct observations were made by walking around the study site and by sitting next to fruiting patches to observe the behavior of potential fruit visitors, in July 2015. The total period of direct observation was approximately 30 h, covering the time between sunset and sunrise, because a preliminary investigation indicated that fruit consumption occurred primarily at night. In addition, consumers of *A. nipponica* fruit were also investigated using the interval‐programming function of a waterproof digital camera (Optio WG40; Pentax, Japan) from July to August 2019. In front of each fruiting individual, a single time‐lapse camera was set to acquire photographs at 50 s intervals, because a direct observation revealed that both crickets and camel crickets typically spent several minutes feeding within a single patch. The cameras captured pictures for approximately 12 h and covered the time between sunset (19:00) and sunrise (5:30), but the observation period varied slightly depending on camera conditions. In total, 41,429 photographs were captured over 575.40 h of monitoring. Only the species that were observed consuming fruits were designated as fruit consumers in the 2015 and 2019 studies.

### SEED VIABILITY

Three individuals of the cricket *Eulandrevus ivani* and three individuals of the camel cricket *Diestrammena yakumontana* were captured after they had consumed the fruits of *A. nipponica* in the field site. After capture, they were kept in separate enclosures and excrements were collected after 48 h, they were examined under a dissection microscope, and the number of intact seeds were counted. The intact seeds were subsequently tested by TTC viability staining, as previously described for dust‐like seeds (de Vega et al. 2011). The viability of defecated seeds was compared with that of the same number of seeds collected directly from the fruit. The differences in viability between seeds from fruits and excrements were assessed using a generalized linear model with a binomial error structure and a logit link. The statistical analysis was performed using R software version 3.6.0 (R Development Core Team 2019).

### FRUIT AND SEED ANATOMY

Fruit samples were fixed in formalin‐acetic acid‐alcohol, dehydrated using an ethanol series, and then embedded in Technovit 7100 resin (Kulzer, Wertheim, Germany) for microtome sectioning. Serial resin sections were cut at a thickness of 4‐5 μm, stained with Safranin O embedded in an Entellan mounting medium (Merck, Darmstadt, Germany), and examined under an Olympus BX‐51 microscope (Olympus, Tokyo, Japan). Using this staining technique, lignified tissues and secondary cell walls were stained red (Zhong and Ye 2007).

## Results

**Figure 2:**
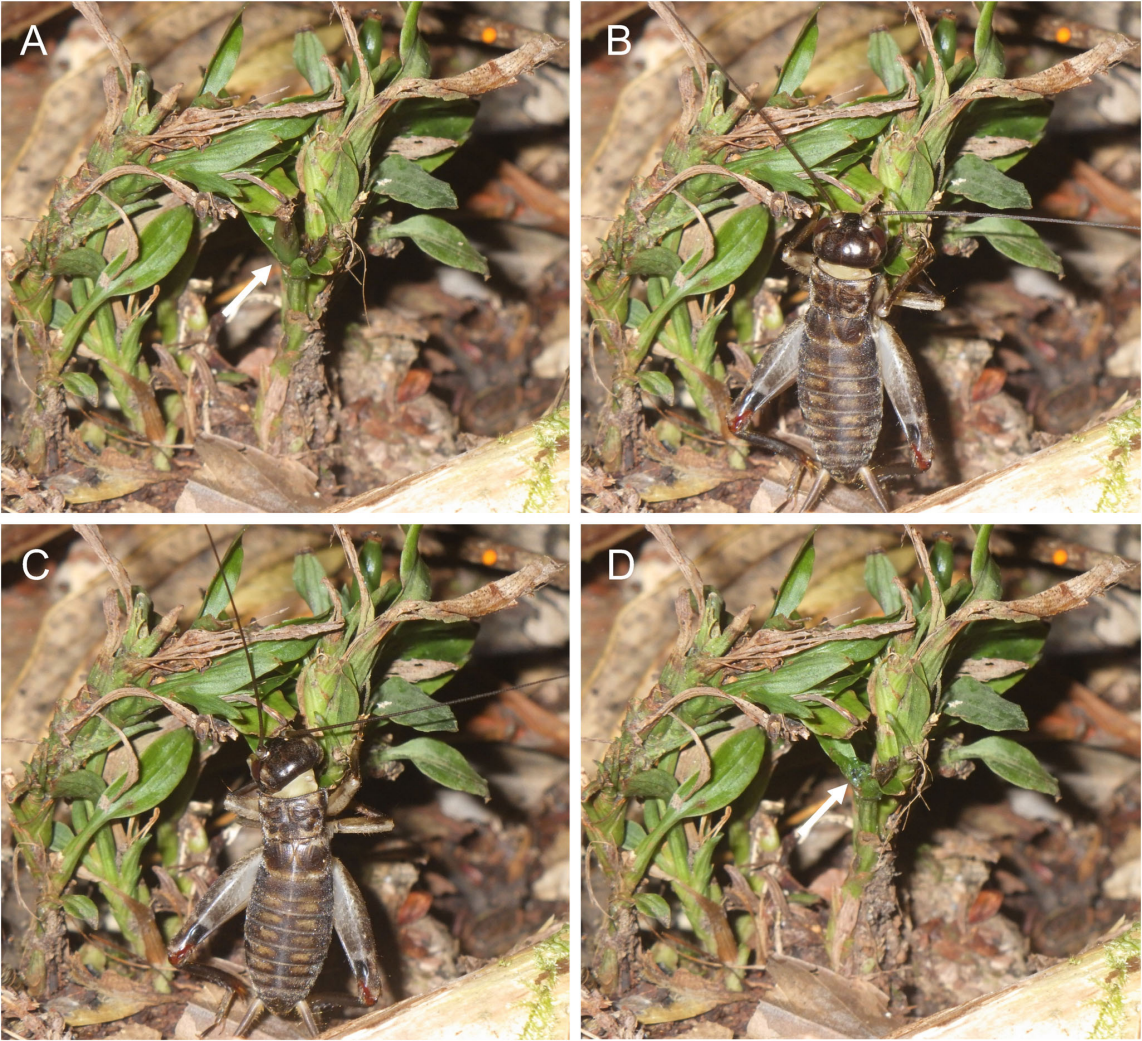
Sequential photographs of the cricket *Eulandrevus ivani* consuming an *Apostasia nipponica* fruit (indicated by arrows). Photos were obtained using time‐lapse photography.

Both direct observation and time‐lapse photography revealed that the fruits of *Apostasia nipponica* were consumed by the cricket, *Eulandrevus ivani*, and the camel cricket, *Diestrammena yakumontana* (Figs. 2 and S1; Table 1). In addition, direct observations showed that the field mouse, *Apodemus argenteus*, completely ignored the *A. nipponica* fruits while in their vicinity. In some cases, the entire fruits were consumed during a single visit by a cricket or camel cricket (Fig. 2). Although it might be possible to detect other foragers by setting the cameras to a shorter frame interval, I consider this to be unlikely, because no additional feeding marks were left on the fruits between the frames with orthopteran visitors. The possibility of water dispersal was also excluded, because no decaying fruits were observed during the study period; rather, the crickets or camel crickets consumed almost all the mature fruits. In particular, time‐lapse photography revealed that, among the nine fruits monitored, six were entirely consumed by crickets or camel crickets. In addition, among the other three fruits, two disappeared despite not showing any signs of decay during the period when the digital camera was not set. Therefore, they were also likely consumed by animals, whereas Apostasioideae seeds have sometimes been considered to be dispersed by water when their indehiscent fruits decay (Wood 1999). The remaining one fruit was left intact. Therefore, seed dispersal by cricket and camel cricket is the dominant seed dispersal system of *A. nipponica* in the investigated population.

**Table 1:** The orthopteran species involved in seed dispersal, and total number of fruit visitations, of *Apostasia nipponica*

Orthopteran species	Times visited (2015)	Times visited (2019)
*Eulandrevus ivani*	4	3 (8)
*Diestrammena yakumontana*	3	9 (24)

In time‐lapse photography (2019), numbers and numbers in parentheses are individuals that fed on the fruits and total numbers of frames captured orthopteran visitors, respectively. The orthopteran visitors were regarded as the same individual when captured in consecutive frames.

All crickets and camel crickets collected during fruit consumption defecated *A. nipponica* seeds in their excrements; each excrement of crickets and camel crickets contained 14.5 ± 9.5 and 10.1 ± 6.5 (*n* = 20 and *n* = 10, mean ± SD) seeds, respectively. Each individual defecated 96.3 ± 80.6 and 33.7 ± 13.3 seeds within 48 h. Microscopic observation revealed that the seeds recovered from the feces of camel cricket remained intact, with normal (i.e., not deformed) embryos, similar to the samples collected from intact fruits. In addition, TTC staining confirmed these observations, and no significant difference was detected in the viability of seeds defecated by crickets (21.4% ± 6.5%), those defecated by camel crickets (25.7% ± 4.1%), and those collected directly from intact fruits (22.6% ± 6.0%). Microtome sectioning confirmed that *A. nipponica* seeds were embedded in the fleshy pulp, which likely facilitates the ingestion of seeds by crickets or camel crickets when they feed on the fleshy pulp. In addition, Safranin O staining showed that the seeds of *A. nipponica* possess a thickened lignified testa that is absent in the dust‐like seeds of most orchids (Fig. 1). The thickened lignified tissue that coats its seeds is probably an adaptation to protect the seeds from digestion by the fruit consumers.

## Discussion

Seed dispersal by animals is a complex mutualistic interaction involving a great diversity of plant and animal species (Howe and Smallwood 1982; Chen et al. 2018). However, the importance of seed dispersal by invertebrates, with the exception of ants, has received comparatively little attention (Bronstein et al. 2006; de Vega et al. 2011). Therefore, discoveries of uncommon mechanisms of seed dispersal by invertebrates such as wetas, beetles, cockroaches, camel crickets, and slugs usually evoke public curiosity toward animal‐plant mutualisms (Duthie et al. 2006; Midgley et al. 2015; Uehara and Sugiura 2017; Chen et al. 2018; Suetsugu 2018a,b).

This study revealed a previously undocumented seed dispersal system in *A. nipponica*, in which its orthopteran visitors consume fruits and excrete viable seeds. The interaction is probably stable at least in the investigated site, because similar results were obtained in different years. The results suggest a stable interaction that constitutes a mutualism, wherein both partners benefit from the association—orthopteran visitors obtain nutrients from the pulp and *A. nipponica* achieves dispersal of seeds from the parent plant (de Vega et al. 2011). The seeds of *A. nipponica* are coated with lignified tissue that in all likelihood protects the seeds as they pass through the digestive tract of crickets and camel crickets. Although neither the cricket nor camel cricket can fly, they potentially transport the seeds long distances owing to their remarkable jumping abilities (Heads and Martins‐Neto 2007). Despite the traditional view that the minute, dust‐like, and wind‐dispersed orchid seeds can travel long distances, both genetic and experimental researches have indicated that orchids have limited dispersal ability; orchid seeds often fall close to the maternal plant (within a few meters), particularly in understory species (Chung et al. 2004; Trapnell et al. 2004; Brzosko et al. 2017). Given that *A. nipponica* fruits are produced close to the ground in dark understory environments, seed dispersal by crickets is probably a successful strategy in *A. nipponica*, occurring under closed canopies where the wind speed is low (Givnish et al. 2005).

With more than 25,700 extant species, the Orthoptera is the most diverse order among the polyneopteran insect lineages (Song et al. 2015). Orthopteran insects occupy every conceivable terrestrial habitat outside the Polar Regions and play integral roles in ecosystems. Because orthopterans are often considered to be detrimental to plants, their participation in mutualistic interactions, such as seed dispersal, has rarely been explored. However, crickets and camel crickets, which are abundant in subtropical and tropical forests, are commonly attracted to fruits on the forest floor (Santana et al. 2016; Suetsugu 2018a,b). It has been shown that crickets consume the arils of arillate seeds and abandon the seeds in other locations, thereby acting as secondary epizoochorous seed dispersers (Santana et al. 2016). These recent discoveries suggest that mutualistic systems involving unexpected taxa might be more common than previously thought.

Curiously, camel crickets are also seed dispersers of several other orchids such as *Yoania amagiensis* and *Yoania japonica* (Suetsugu 2018a,b). This is noteworthy because all orchid seeds lack endosperms; their embryos contain only marginal carbon reserves, owing to their initial mycoheterotrophy (Zhang et al. 2017). Orchids increase the chances of encounters with host fungi by minimizing maternal investment in individual seeds while maximizing the number of seeds (Eriksson and Kainulainen 2011). Endozoochory can occur whenever seeds are swallowed whole and are resilient enough to remain intact after passage through the gut of animals (de Vega et al. 2011; McCormick et al. 2013). Therefore, the small size of orchid seeds enabled by mycoheterotrophic germination may be a predisposition to the evolution of endozoochory by small animals. Whether seed dispersal by orthopterans is common in the members of Apostasioideae remains to be investigated. Nonetheless, the gross fruit morphology and pigmentation pattern of some *Apostasia* species, such as *Apostasia shenzhenica* and *Apostasia fogangica*, parallel those seen in *A. nipponica* (Chen and Liu 2011; Yin et al. 2016), suggesting that similar seed dispersal systems could be widespread among these species.

Despite being the most diverse plant family, many aspects of the evolutionary history of Orchidaceae remain obscure. In particular, because of their extremely minute size, the orchid seeds lack a definitive fossil record (Gołaszewska et al. 2019). Therefore, the interaction described here provides some clues regarding the animals that may have participated in the seed dispersal of ancestral clades of orchids. The family Gryllidae (crickets) represents early diverging clades within Orthoptera and is considered to have separated from other groups in the Triassic, approximately 240 million years ago, whereas the family Rhaphidophoridae (camel crickets) is thought to have originated approximately 140 million years ago, based on molecular studies (Song et al. 2015). In contrast, orchids appear to have diverged from the common ancestor of all other members of Asparagales approximately 112 million years ago (Givnish et al., 2015, 2016). Therefore, both crickets and camel crickets were arguably available when orchids originated, and they are among the candidates for seed dispersers of the ancestor of extant orchids.

Owing to many plesiomorphic characters and the earliest diverging phylogenetic position, members of Apostasioideae have been extensively studied to understand their floral structure, taxonomy, biogeography, and genome (Kocyan and Endress 2001; Chen and Liu 2011; Niu et al. 2017; Zhang et al. 2017). Hoverer, there is still a lack of information regarding seed dispersal in the subfamily. Here, I describe seed dispersal of Apostasioideae members by animals for the first time. Whether seed dispersal by animals (and particularly by orthopteran fruit feeders) is common in these orchids warrants further investigation. Although zoochory has also evolved secondarily from wind dispersal, at least twice within other orchid subfamilies (Suetsugu et al. 2015; Suetsugu 2018a,b), it is even possible that animal‐mediated seed dispersal is an ancestral trait in Apostasioideae, given that indehiscent fruits with hard seed coat are common within the clade (Molvray and Chase 1999). Ancestral character‐state reconstruction analysis, with more data on the seed dispersal systems of other apostasioids, will provide deeper insights into the early evolution of the seed dispersal system in Orchidaceae.

## AUTHOR CONTRIBUTIONS

KS conceived and designed the study, conducted field study and laboratory experiments, and wrote the manuscript.

## DATA ARCHIVING

The data that support the findings of this study are available from the corresponding author on request.

## CONFLICT OF INTEREST

The author declares no conflict of interest.

**Associate Editor: C. Moreau**

## Supporting information


**Figure S1**. Sequential photographs of the camel cricket *Diestrammena yakumontana* consuming an *Apostasia nipponica* fruit (indicated by arrows).

**Table S1**. Raw data of the number of intact seeds and viable seeds defecated by the cricket and camel cricket collected in its natural habitat during fruit consumption.
**Table S2**. The number of intact seeds in each excrement defecated by the cricket and camel cricket collected in its natural habitat during fruit consumption.
