# Supplementary figures and images for: A novel seed dispersal mode of Apostasia nipponica could provide some clues to the early evolution of the seed dispersal system in Orchidaceae
Source: Evol Lett. 2020 Aug 2;4(5):457–64. doi: 10.1002/evl3.188 (PMC7523560; doi:10.1002/evl3.188)

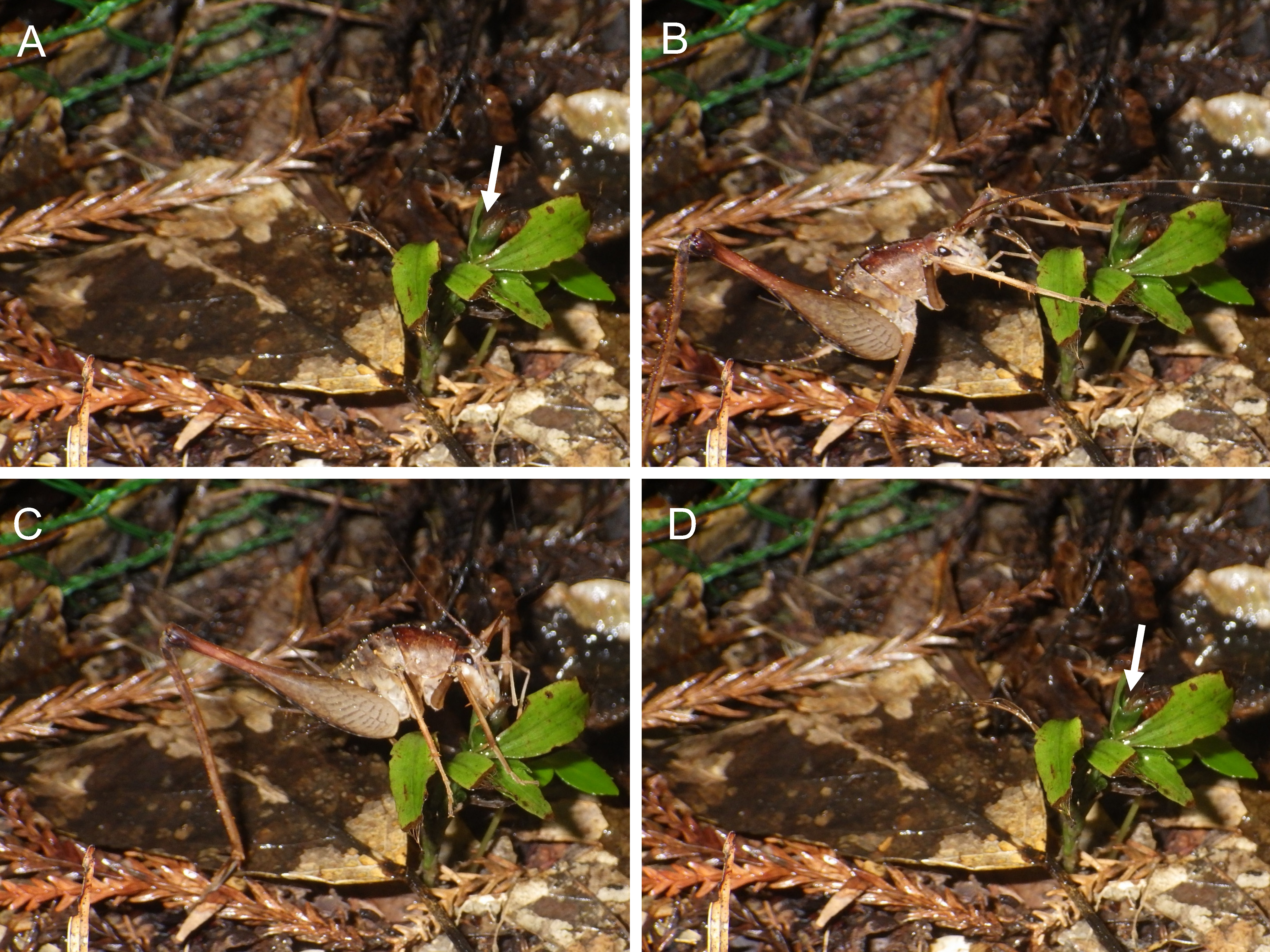

Supplement: Supplementary file 1 — Figure S1. Sequential photographs of the camel cricket Diestrammena yakumontana consuming an Apostasia nipponica fruit (indicated by arrows). [file EVL3-4-457-s001.jpg]
